# Squamous Cell Carcinoma Arising within Verruca Vulgaris on the Nipple

**DOI:** 10.1155/2018/1984593

**Published:** 2018-06-20

**Authors:** Araya Zaesim, Amanda C. Jackson, Sang Wook Lee, Shaun A. Price

**Affiliations:** ^1^Mercer University School of Medicine, Columbus, GA, USA; ^2^Department of General Surgery, St. Francis Center for Surgical Care, Columbus, GA, USA

## Abstract

Cutaneous squamous cell carcinoma (SCC) is a common form of skin cancer and often appears as a hard, scaly lump that occasionally ulcerates. It is usually associated with cumulative exposure to ultraviolet light, although prior scarring, chronic wounds, exposure to radiation, HPV infection, and immunosuppression are also associated risk factors. Primary SCC of the nipple is very rare and only a few cases have been reported. We present a case of a 49-year-old female with concerns of a right nipple lesion with erythema and pain. She was initially evaluated for Paget's disease with an underlying malignancy and cellulitis, but, after biopsy and investigation, she was found to have a well-differentiated SCC arising from a verruca vulgaris. Current literature does not provide distinct guidelines on management of SCC or its variants on the nipple, and the case was managed based off of SCC at other cutaneous sites as well as other cases of SCC on the nipple.

## 1. Introduction

Squamous cell carcinoma (SCC) of the skin and basal cell carcinoma of the skin are the most common cancers diagnosed annually in the United States [1]. Exact numbers are unknown as these common nonmelanoma skin cancers are not required to be reported to cancer registries. SCC arises from malignant transformation of epidermal keratinocytes. Examination of a suspected lesion typically reveals a hard and scaly plaque or nodule that occasionally ulcerates [2]. The most common reported risk factor for SCC is cumulative exposure to ultraviolet light [3]. Other risk factors include scarring, chronic wounds, ionizing radiation, HPV infection, and immunosuppression. Cutaneous SCC is usually not fatal, but it is associated with invasion causing local disfigurement and occasionally distant metastases. Primary SCC of the nipple is extremely rare and is noted in English literature only ten times [4–13]. Management of SCC of the nipple is derived from the understanding of cutaneous SCC in more common areas, although a wide variety of treatment modalities has been reported.

## 2. Case Report

A 49-year-old Caucasian woman was referred to a general surgeon by her primary care physician for a right nipple lesion. She reports no past medical history, and social history is notable for tobacco use. The patient stated that she first noticed a small, yellow, and fleshy bump on her right nipple approximately two years prior. She became concerned due to its rapid growth in the last year. Four months prior, she visited her primary care provider who performed a shave biopsy of the lesion. Initial pathology results suggested squamous cell carcinoma, but this was thought to be discordant with the clinical picture. At consultation, she reported that her right breast had also started feeling hot and tender for two weeks duration. On examination, her right breast was erythematous and rigid with a 2.2 cm lesion consuming the right nipple. Her nipple also drained yellow pus. The patient was placed on a course of antibiotics due to concerns for an abscess.

**Figure 1:**
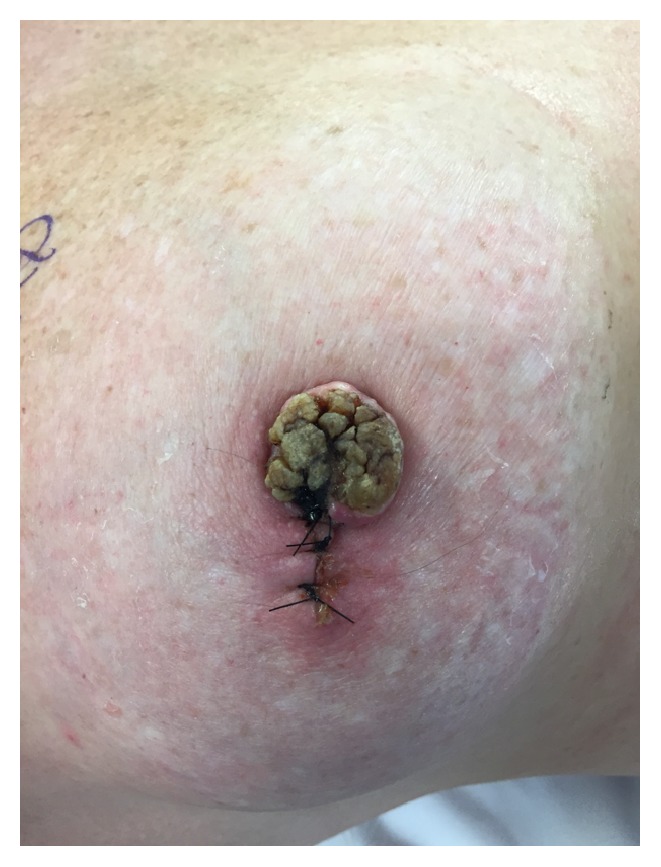
A wedge biopsy was taken from the nipple which appeared to have a 2.2 cm lesion.

An MRI was ordered to investigate possible underlying breast malignancy. Results of the MRI showed no solid mass underlying the nipple. A wedge biopsy of the nipple was performed to confirm the initial shave biopsy pathology (Figure 1). Pathology showed verrucous histologic features with chronic inflammation and underlying abscess with concerns for a possible cutaneous malignancy. Specifically, there was marked papillomatosis with hypergranulomatosis between the papillae as well as a lack of granules at the papillary surfaces. Immunohistochemical testing was also positive for HPV L1 capsid protein. Because the initial shave biopsy showed SCC with positive margins, this clinical picture suggested that an inadequate sample was taken during the wedge biopsy. After consultation of literature, excision with wide margins was determined to be appropriate for removal of the lesion.

**Figure 2:**
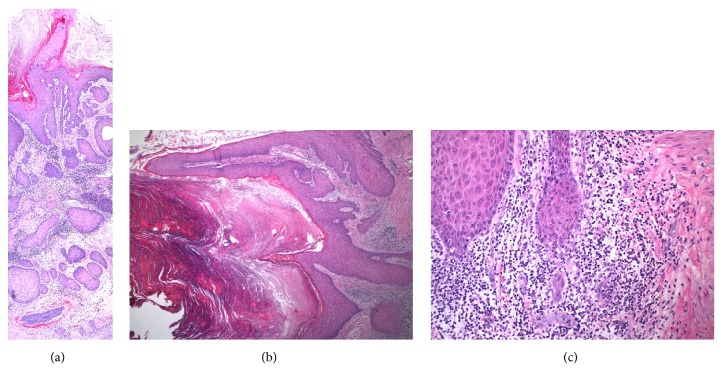
Histologic examination of a large section of tissue at 10x magnification (a) demonstrates the verrucoid top of the lesion and the invasive deep edge. Wide nests of tumor cells can be seen invading as well as a desmoplastic reaction to the nests and the lymphoplasmacytic host response. A low power image of the verrucous surface at 4x magnification (b) shows the verrucous surface with peaking hyperkeratosis and subtle koilocytotic changes. A high power image at 20x (c) shows dissolution of the basal lamina and invasion of the dermis. There is invasion by a small cluster of keratinocytes with the expected lymphoplasmacytic host response and desmoplastic host response (samples stained with hematoxylin and eosin).

The patient underwent a wide excision of the right nipple-areola complex for removal of the growth. A 6.2 cm x 3.2 cm skin ellipse was excised encompassing the 2.2 cm nipple lesion. On final histologic examination, the lesion was found to be a large tumor with verrucous features, hyperkeratosis, dyskeratosis, and nests of keratin pearl formation (Figure 2). Two areas showed microinvasion of the dermis with loss of the basal lamina. There were also significant acute and chronic inflammatory responses in the deep dermis representing an underlying abscess. These findings are congruent with the diagnosis of a well-differentiated squamous cell carcinoma with arising within verruca vulgaris. All margins were found to be clear of tumor cells. The post-op course was uneventful. She continues to follow up to monitor potential recurrence of the growth.

## 3. Discussion

Although cutaneous SCC is a very common diagnosis, to have it occur on the nipple is unlikely. This is primarily due to risk factors associated with the disease. They are usually associated with cumulative exposure to ultraviolet light although prior scarring, chronic wounds, exposure to radiation, HPV infection, smoking, and immunosuppression are also associated risk factors. Ultraviolet light exposure is by far the most important risk factor [3]. Thus, in a location such as the nipple which does not see as much cumulative lifetime ultraviolet exposure as other skin locations, it is no wonder that the occurrence of SCC there is rare.

In the case of the presented patient, minor sun damage to the upper chest was noted. The patient also reported infrequent use of tanning beds, which could have induced pathology in the presenting location. However, she has never had any previous history of skin pathology to date. It would be expected that this patient presenting with a nonmelanoma skin cancer would have had pathology in areas with more cumulative ultraviolet exposure such as the face, hands, arms, and upper chest. Of previously reported cases of cutaneous SCC of the nipple, most patients had previous history of multiple nonmelanoma skin cancers in other locations. It is strange that this patient's presentation of her first diagnosed nonmelanoma skin cancer was on the nipple-areola complex. Patients with their first nonmelanoma skin cancer have a 3-year mean cumulative risk of 47% for development of another nonmelanoma skin cancer highlighting the need for adequate follow-up and monitoring [14].

The patient presented represents a unique instance of SCC of the nipple. No previous literature accounts for SCC of the nipple arising within a biopsy proven verruca vulgaris. Verruca vulgaris, or more commonly warts, is primarily caused by viruses within the family of human papilloma viruses (HPV). HPV is a group of approximately 100 strains of viruses that can cause disordered epithelial growth. This often results in the growth of warts on the hands, plantar surface of the feet, or anogenital region [15]. HPV strains are well-recognized contributors to SCC arising in anogenital, penile, cervical, and oropharyngeal areas of the body. The mutagenic properties of HPV are due to encoded viral oncoproteins that inhibit host tumor suppressor genes [16]. Immunosuppressive disorders, sun exposure, and tobacco use increase risk of HPV-related SCC. The presented patient is immunocompetent and has no prior history of HPV-related pathologies (i.e., abnormal pap smear, anogenital warts, etc.). However, chronic tobacco use combined with HPV exposure could have increased her risk of developing SCC. Literature searches reveal that while HPV may increase risk of SCC, it is still quite rare to have a verruca vulgaris arise from the nipple let alone transform into cutaneous SCC.

Although SCC can arise de novo and contain verrucoid features, confidence regarding the diagnosis of verruca vulgaris is gained from the initial biopsy at the lesion edge which strongly appeared as a verruca vulgaris and stained positively for an HPV capsid protein. Also, the patient's history of an initial, fleshy lesion on the nipple must be taken into account as a verruca vulgaris is often diagnosed by patient history.

One major differential diagnosis physicians must consider when a patient presents with a lesion of the nipple is Paget's disease of the breast (PDB). PDB is an uncommon diagnosis, but it is the most common lesion of the nipple [17]. It is crucial that this diagnosis be ruled out as PDB is often associated with an underlying breast cancer [18, 19]. Described as being a thickened, crusted lesion with irregular borders usually limited to the nipple-areola complex, it can be hard to visually distinguish from a possible SCC. Suggested management of PDB includes an initial biopsy which can confirm the lesion or lead to an alternative diagnosis such as SCC. Guidelines also suggest further investigation using a mammogram or MRI to rule out any underlying breast malignancy. Such a distinction is important as it can guide differing management techniques.

As SCC of the nipple has a very low incidence, no standard management guidelines have been established. Many of the current examples in literature suggest wide local excision as the primary mode of treatment, but some patients have been offered mastectomy and radiation [4–13]. Two of the cases reported local recurrence, of which one had local resection with unclear margins. The other case was treated with novel photodynamic therapy with cryotherapy. With surgical resection, as with resection of cutaneous carcinomas on other areas of the skin, clear margins provide the best prognosis. One case which was treated with mastectomy was last reported as having no recurrence at the five-year point [7]. More data is still needed to determine the best mode of treatment.

## Data Availability

Readers may contact the corresponding author for any requests for data used in the completion of this manuscript.
